# Insufficientia Pylori

**Published:** 1904-05-28

**Authors:** 


### INSUFFICIENTIA PYLORI.

This condition, which is thought to follow upon prolonged irritation of the pylorus, is, according to Dr. Mark I. Knapp,[1] one of the most important causes of chronic dyspepsia, and one, moreover, which it is essential to recognise, as treatment is in most cases very successful. The condition, he says, cannot be diagnosed without using test-meals. Absence of chyme in the stomach one hour after a test-breakfast is the diagnostic criterion. Normally the stomach should yield from 30 to 50 c.c. of chyme one hour after Ewald's test-breakfast, the solid part of which should be well pulverised, and the fluid part quite thin. The chyme should readily filter. If nothing or only one or two cubic centimetres of chyme can be aspirated one hour after the test-meal, insufficiency of the pylorus is present.

The condition is due to excessive stimulation of the pyloric muscle. The degree of contraction of the stomach walls in response to food depends upon the nature of the ingested material. Improperly masticated or irritating food will cause excessive contraction of the muscular coats, and this overactivity will involve the pylorus equally with the rest of the stomach; but while the stomach walls have a considerable lumen to contract upon, the pylorus has nothing more than its own fibres on which to contract. Further, the food particles at each contraction will be pressed forcibly against the pylorus. The consequence of such repeated pressure is inflammation, and eventual loss of function of the pyloric muscle fibres. So commonly is the pylorus the seat of lesion in chronic disease of the stomach, that it would be justifiable, Dr. Knapp believes, to discard the name of gastritis in the majority of instances and substitute the name of pyloritis instead. The muscular activity of the stomach is an important function analogous to that of the gizzard in birds, and on it depends the fine pulverisation of food during gastric digestion, a phenomenon which does not occur in artificial digestion in vitro. Clinical experience shows that this trituration of food is mainly independent of the amount of acid present in the stomach contents. It is therefore apparent that to obtain a very fine pulverisation of food it must be subjected for a sufficiently long time to the gastric movements, and hence the aspiration from the stomach of much finely divided food in the form of chyme is evidence of normal if not excessive motility of the stomach. This is the reverse of the usual teaching that a stomach which yields a large quantity of chyme after a test-breakfast is atonic. On the other hand, a pylorus, the activity of which is lessened or entirely abolished, offers little or no resistance to the exit of the stomach contents, hence the latter will pass more easily and quickly into the intestine. The absence of stomach contents one hour after a test-meal cannot be attributed to hypermotility, as the latter must be accompanied by overactivity of the pylorus. The true explanation in such a case is deficient contraction of the pyloric sphincter. Very little peristalsis is necessary to bring ingesta past an open pylorus, mere gravity will be sufficient.

Assuming that the above explanation is the correct one, insufficiency of the pylorus is consequent upon inflammation secondary to hyperacidity of the stomach contents, chronic alcoholism, loss of teeth, deficient mastication, and improper diet. So far as prognosis is concerned, it is excellent in the absence of severe complications, no matter how old the patient is or how long the defect has existed, although a return of the pylorus to the normal state can hardly be expected except in the young. Treatment will be guided by a knowledge of the etiological factor in each individual case; alcohol must be interdicted, deficient teeth supplanted by artificial ones, proper mastication insisted on, all cereals and vegetables well boiled and eaten in the form of puree, and all indigestible food strictly avoided.

[1] Medical Record, April 30.

